# Comparing the Outcomes of Digital and Traditional Cardiac Rehabilitation Practices: A Systematic Review and Meta-Analysis

**DOI:** 10.7759/cureus.77757

**Published:** 2025-01-21

**Authors:** Sumbul Ansari, Bhuvaneshwari G Nadar, M. Dulce Estêvão, Débora R Aguiar, Jude Ejeh, Zahid Khan

**Affiliations:** 1 Department of Physiotherapy, School of Allied Health Sciences, Galgotias University, Greater Noida, IND; 2 Public Health Dentistry, Terna Dental College and Hospital, Mumbai, IND; 3 School of Health, University of Algarve, Faro, PRT; 4 Algarve Biomedical Center Research Institute, University of Algarve, Faro, PRT; 5 Escola Paulista de Medicina, Universidade Federal de São Paulo, São Paulo, BRA; 6 Faculty of Medicine, University of Geneva/Hôpital de la Tour, Geneva, CHE; 7 Cardiology, University of South Wales, Pontypridd, GBR; 8 Cardiology, University of Buckingham, London, GBR; 9 Cardiology, Bart’s Heart Centre, London, GBR

**Keywords:** center-based cardiac rehab, centre based cardiac rehabilitation, cost-effectiveness analysis, digital cardiac rehabilitation, home-based cardiac rehab, major adverse cardiac events (mace), major adverse cardiovascular events (mace), quality of life (qol), rehospitalisation, systematic review and meta-analysis

## Abstract

This systematic review and meta-analysis aimed to evaluate the effects of digital cardiac rehabilitation (DCR) encompassing application-based telehealth compared to traditional cardiac rehabilitation onmajor adverse cardiovascular events (MACE), rehospitalisation, costs, quality of life (QoL), and physical activity levels in patients with coronary artery disease (CAD).

From 2014 to May 2024, a systematic search of the MEDLINE, PubMed, Web of Science, and Scopus databases was conducted using relevant keywords to identify randomised controlled trials (RCTs) or randomised cross-over trials. The methodological quality of the included studies was assessed using the Physiotherapy Evidence Database (PEDro) scale and risk of bias tool. The included articles were then subjected to qualitative synthesis and meta-analysis.

Thirteen studies involving 1850 participants were included in the study. Meta-analysis revealed statistically significant improvements in QoL (mean deviation (MD) = 0.10, 95% CI: 0.05-0.15, p = 0.0002). DCR compared with centre-based rehabilitation (CBR). These improvements in QoL likely translated to enhanced daily functioning, such as the increased ability to perform activities of daily living. However, no significant differences were found for physical activity levels (MD = 1.69, 95% CI: 1.49-4.87, p = 0.30), rehospitalisation (relative risk (RR) = 0.86, 95% CI: 0.66-1.11, p = 0.25) or MACE (RR = 0.67, 95% CI: 0.42-1.07, p = 0.09). High heterogeneity was observed in QoL, likely due to variations in DCR modalities, study populations, and intervention content. The results of this study, therefore, must be interpreted with caution.

DCR may offer significant benefits in terms of improving the QoL in patients with CAD. While promising trends were observed for rehospitalisation and MACE, further research is needed to confirm these findings. Potential reasons for the observed benefits of DCR over centre-based rehabilitation plausibly include improved accessibility, enhanced patient engagement, and greater flexibility. However, it is important to acknowledge the presence of heterogeneity among the included studies and potential gender imbalances within the study populations, which may have influenced the results. Future research should prioritize long-term outcomes, cost-effectiveness, real-world effectiveness in diverse populations, and the development of standardized DCR protocols.

## Introduction and background

Cardiovascular diseases (CVD) are heart and blood vessel diseases with considerable morbidity and mortality and present a worldwide public health burden. Moreover, CVDs are the leading cause of death globally, accounting for 31% of all global deaths [1]. Risk factors, including body mass index (BMI), blood pressure, cholesterol, smoking, and diabetes, contribute significantly to the burden of cardiovascular disease. However, the precise proportion attributable to these factors varies depending on the population and research methodology. CVD constitutes a major source of financial hardship, resulting in financial distress. This can manifest in various forms, such as psychological distress, cost-related non-adherence to treatment regimens, deferral of essential medical care, and the prioritization of healthcare expenses over basic living needs.

Cardiovascular disease is the leading cause of mortality and morbidity worldwide [1]. Coronary artery disease (CAD) is a major contributor to the high mortality rate [2]. CAD occurs when the coronary arteries, which supply blood to the heart, become narrowed or blocked owing to atherosclerosis. This can lead to heart muscle damage and ultimately death [3]. CAD is a significant cause of disability and death in both developed and developing nations [4]. Despite advancements in medical therapies, lifestyle modifications, and surgical interventions, CAD continues to pose a significant burden on the healthcare system.

To address this significant health issue, cardiac rehabilitation (CR), a multifaceted approach that combines various therapies, such as risk factor education, behavior modification, psychological and vocational support, and nutritional counseling, seems to be promising. These interventions focus on reducing the risk factors for CAD [5], with physical activity and exercise forming the core components, constituting 30-70% of the program. Exercise training has been demonstrated to directly benefit the heart and blood vessels by improving myocardial oxygen demand, endothelial function, autonomic nervous system activity, blood clotting, inflammation, and the development of collateral blood vessels. While these direct effects are important, CR may also reduce mortality by indirectly improving traditional risk factors for CVD, such as cholesterol levels, smoking cessation, and blood pressure control. CR programs typically incorporate a diverse range of exercise modalities tailored to individual needs and conditions. Aerobic exercises, such as walking, cycling, swimming, and rowing, are fundamental, aiming to enhance cardiovascular fitness. Strength training, including weightlifting and bodyweight exercises, is crucial for building muscle mass and improving overall strength. Furthermore, flexibility and balance exercises, such as yoga, tai chi, and stretching, are incorporated to improve range of motion, coordination, and stability. CR is recommended by the European Society of Cardiology guidelines (class IA) [6] for patients with CAD because of its association with reduced cardiovascular mortality and morbidity, improved quality of life (QoL) [7,8], and significant healthcare cost reduction [9].

Participation in traditional centre-based CR programs remains low [10] despite efforts to increase utilisation. Traditional in-person CR programs often face barriers, such as limited accessibility, time constraints, and transportation difficulties. Rising healthcare costs have limited the expansion of CR programs, necessitating the development of alternative care strategies. Advancements in telecommunication technology have led to the development of cardiac telerehabilitation programs, enabling remote patient rehabilitation through telemonitoring, telecoaching, and e-learning. In recent years, digital health technologies have emerged as promising solutions to address these challenges. Digital cardiac rehabilitation (DCR) programs offer a convenient and flexible approach to delivering evidence-based interventions to patients with CAD [11]. DCR leverages digital technologies, such as the internet, smartphone applications, phone calls, and video conferencing, to extend the reach of CR beyond traditional in-person settings. This approach enables patients to receive essential support, including regular feedback, personalized coaching, and expert consultations, within the comfort of their homes. While small-scale studies and recent systematic reviews [12] suggest cardiac telerehabilitation's cost-effectiveness compared to centre-based CR, larger-scale studies and comprehensive cost-effectiveness analyses are still required [13]. Despite the established clinical benefits of traditional centre-based CR programs [14], long-term outcomes are often underwhelming due to low participation and adherence rates [15]. The positive impact of mobile health systems has been observed in primary prevention patients at high risk of cardiovascular disease [16-18]. A recent systematic review involving 2250 participants analysing cardiac telerehabilitation programs concluded that these strategies could enhance the utilisation and reach of CR programs [13].

Meta-analysis data [19] and a prior pilot study on a separate but similar cohort of non-randomised patients demonstrated benefits in intermediate markers of secondary CVD prevention as well as rehospitalisations and emergency department (ED) visits in patients who were prescribed digital health interventions for DCR [20]. DCR has proven cost-effective compared to no intervention, even in the era of advanced medical therapy and coronary revascularization [21]. Two recent systematic reviews have shown the non-inferiority of DCR to centre-based CR in terms of feasibility, safety, and effectiveness [14,22]. Similarly, recent meta-analyses of clinical trials in patients with CAD have indicated that secondary prevention programs incorporating telehealth interventions, either independently or combined with on-site CR, may lead to reductions in adverse events, lifestyle improvements, and better control of cardiovascular risk factors(s) control [23,24]. These findings highlight the need for further research to assess the effectiveness of telehealth interventions. However, these earlier reviews [14,22] lacked information on important outcomes, such as rehospitalisation, costs, physical activity, and major adverse cardiovascular events (MACE). MACE data are important for assessing the long-term impact of DCR on patient health and survival, while data on costs are essential for evaluating the value proposition of DCR programs, informing healthcare resource allocation decisions, and facilitating the wider adoption of these interventions within healthcare systems. Additionally, individual studies provide limited evidence, necessitating synthesis to assess the effectiveness of interventions. Reviews and meta-analysis mostly focus on CVD risk factors. To address these research gaps, the present systematic review and meta-analysis aimed to evaluate the impact of DCR on key clinical outcomes, including quality of life, rehospitalisation, major adverse cardiovascular events, physical activity levels, and costs in patients with CAD. By synthesising the most recent available evidence, this study sought to inform clinical practice regarding the effectiveness of DCR.

## Review

Methodology

Registration 

This systematic review and meta-analysis was conducted in accordance with the Preferred Reporting Items for Systematic Reviews and Meta-Analyses (PRISMA) 2020 guidelines [25]. The study protocol was prospectively registered in the International Prospective Register of Systematic Reviews (PROSPERO) (Reg. ID CRD42024551745). 

*Eligibility Criteria*****

To be eligible for inclusion in this review, studies were required to be RCTs or randomised cross-over studies conducted on patients diagnosed with CAD, published in the English language, and to compare outcomes such as hospital readmission, mortality, healthcare costs, physical activity levels, and QoL to a control or comparison group. This study was limited to English-language publications. This restriction may have introduced language bias, as studies conducted in other languages may not have been included in the search. Furthermore, grey literature and conference abstracts were excluded from the search. These sources were omitted primarily due to concerns about their quality and completeness. Grey literature can be difficult to assess for methodological rigor and may lack the peer-review process that ensures scientific validity. Similarly, conference abstracts often provide preliminary findings and may not contain sufficient detail for comprehensive analysis.

Data Sources and Searches 

A comprehensive literature search was conducted in the PubMed, Scopus, and Web of Science databases from January 2014 to May 2024. The search strategy was informed by the Population, Intervention, Comparison, and Outcome (PICO) framework, as outlined in [26]. The PICO components for this review were defined as follows: P: Patients with CAD, I: DCR, C: Control group; O: Hospital readmission; mortality such as MACE, cost, physical activity level, and QoL. The following keywords or combinations were used: (1) “Coronary artery disease” OR “CAD”; (2) “Digital cardiac rehabilitation” OR “Digital rehabilitation” OR “Digital rehab” OR “Telerehabilitation” OR “Telerehab” OR “Virtual rehabilitation”; (3) “Hospital readmissions” OR “Mortality” OR “Costs” OR “Physical activity” OR “Physical activity levels” OR “Quality of life” OR “Major adverse cardiovascular events” OR “MACE” and (4) (1) AND (2), (1) AND (3), (1) AND (2) AND (3) or (1) AND (2) AND (3) AND (4). To enhance the comprehensiveness of the search strategy, a manual search of the reference lists of included studies and relevant systematic reviews was conducted. This process involved meticulously examining the reference sections of all identified studies and extracting relevant citations.

Two independent reviewers screened eligible studies based on predefined inclusion and exclusion criteria. Data extraction was conducted manually by the same reviewers using a standardised Excel form, with a focus on baseline characteristics, intervention details, and outcome measures. The extracted data included author names, publication year, study design, participant demographics (sample size, sex, age, and loss to follow-up), inclusion and exclusion criteria, intervention characteristics (number of groups, frequency, and duration), and key outcomes (hospital readmission, MACE, healthcare costs, physical activity levels, and QoL). A third reviewer verified the extracted data and methodology. 

*Methodological Quality Assessment*****

The methodological quality of the included studies was assessed by using the 11-item Physiotherapy Evidence Database (PEDro) scale [27,28]. Any disagreements were resolved through mutual consensus or arbitration by a third reviewer. The PEDro scale, which incorporates elements from the Jadad scale and Delphi list [29,30], assigns a binary score (0 or 1) to each item. The total score, excluding the "eligibility criteria specified" item, ranged from 0 to 10. Studies were categorised as having poor, fair, good, or excellent quality based on established cut-off scores of <4, 4-5, 6-8, or 9-10, respectively [31]. To ensure consistency in quality assessment, two independent reviewers assessed the methodological quality of each study using the PEDro scale. Discrepancies between reviewers were resolved through discussion and consensus. In cases where consensus could not be reached, a third reviewer was consulted to adjudicate the disagreement.

*Risk of Bias Assessment*****

Two researchers independently assessed the potential biases in the included studies using the Cochrane risk-of-bias tool. This tool categorises bias into three levels: high, low, and unclear. The evaluation covered seven domains: randomisation, allocation concealment, blinding of participants and personnel, blinding of outcome assessment, handling of incomplete data, selective reporting, and other potential biases. Disagreements between the two researchers were resolved through mutual consensus and with input from a third researcher. This ensured that the final decisions were unbiased and reflected a comprehensive evaluation of all perspectives.

Strategies for Data Synthesis

Baseline and post-intervention data for relevant outcome measures were extracted from the included studies for both intervention and control groups. For each outcome, the number of participants (n), mean, standard deviation, p-value, and 95% confidence interval (CI) were recorded. Data were entered into Review Manager (RevMan 5.4, The Cochrane Collaboration) for the meta-analysis. Both fixed and random effects models were employed to calculate the mean differences (MDs)/risk ratio (RR) for the outcome measures (rehospitalisation, MACE, physical activity levels, and QoL), along with their 95% CIs. The magnitude of the treatment effect was interpreted using the following effect-size guidelines: 0.2 (small 0.8), medium (0.5), and 0.8 (large) [32]. Heterogeneity among studies was assessed using the I² statistic with the following interpretation: 0-40% (low), 30-60% (moderate), 50-90% (high), and 75-100% (very high) [33]. A bar graph was generated to visualise the cost data by aggregating the cost information from the studies that reported this variable. Statistical significance was set at P < 0.05.

Results

Study Selection

**Figure 1:**
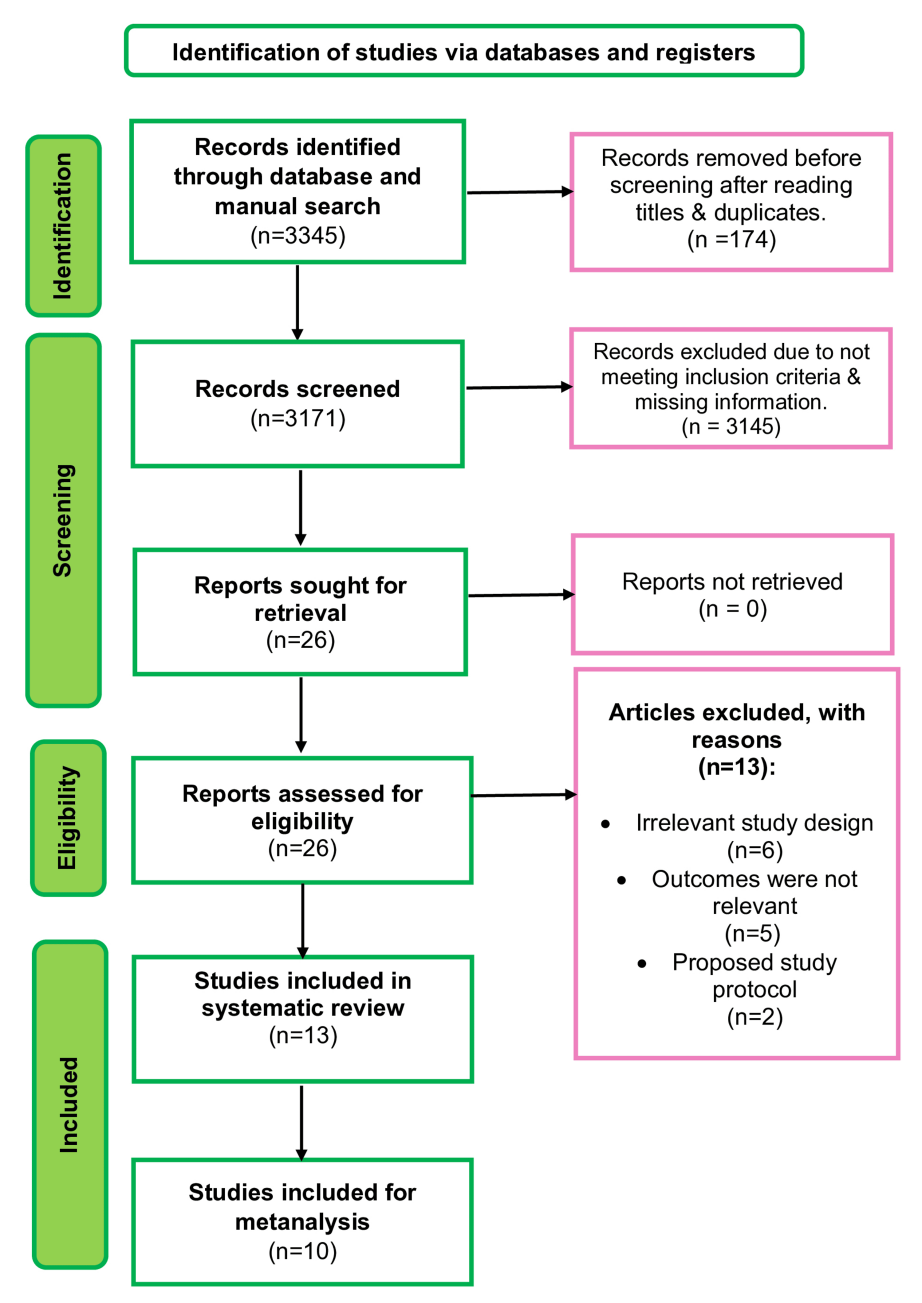
PRISMA diagram for the present study The image shows the breakdown of the studies included in this review as per the Preferred Reporting Items for Systematic Reviews and Meta-Analyses (PRISMA) flow diagram.

Figure 1 presents the PRISMA flow diagram detailing the selection process of the included studies. An initial search of various electronic databases and additional sources identified 3345 articles. After eliminating duplicates, 3171 articles were subjected to title and abstract screening. Subsequently, 26 full-text articles were assessed for their eligibility. Ultimately, 13 articles were excluded because of language barriers, inappropriate study design, or unavailability of full-text versions. The remaining 13 articles [34-46] fulfilled the inclusion criteria and were included in the systematic review and meta-analysis. A total of one additional study was identified through this manual search process.

Population Characteristics

​A total of 1850 individuals with CAD took part in the final 13 studies.​ There were no differences in the gender composition of the participants; all studies included participants of both genders. Among the 1850 participants, 79.3% were male, 19.6% were female, and 1.1% of participants remained unspecified. The mean age reported in the studies varied from a minimum of 55.8 years [40] to a maximum of 66.7 years [38]. The demographic details of the included studies are presented in Table 1.

**Table 1:** Patient demographic characteristics data for studies included this review HTN (Hypertension), T2DM (Type 2 diabetes mellitus), CV (Cardiovascular), QoL (Quality of life), VO2 (Volume of oxygen), PA (Physical activity), HRQL (Health-related quality of life), I (intervention), C (Control), HbA1c (glycated haemoglobin), BHF (British Heart Foundation), N (Number), MACE (Major Adverse Cardiovascular Events), mHealth (Mobile health), 6-MWD (6 minute walking distance), MI (myocardial Infarction), METS (Metabolic equivalents), ED (Emergency department), CBCR (Centre-based cardiac rehabilitation), RCT (Randomised controlled trial), UK (United Kingdom), USA (United States), DHI (Digital health intervention), HBCT (Home-based cardiac rehabilitation), PACES (physical activity after cardiac events).

Authors & Year	Study Type and country	N	Age (Mean, SD)	Comorbidities	Interventions	Outcome measures	Outcome Measures - Intervals	Main findings
Herring et al., 2021 [34]	RCT (UK)	N=291	C:66.6(10.2)	HTN T2DM Myocardial infarction	C: General health advice.	PA, Lipid profile, HbA1c, QoL.	At baseline, 6m, 12m	No significant differences in overall PA; MVPA increased significantly in those not meeting guidelines at enrollment.
			I:66.48(9.2)		I: BHF leaflet + PACES education (2x2.5h sessions) + motivational texts (82 messages).			
Brouwers et al., 2021 [35]	Cost-utility Analysis (Netherlands)	N=300	Mean:60.7(9.5)	T2DM, HTN, hypercholesterolemia.	C: Group-based exercise;	QoL, costs, rehospitalizations	Follow-up assessments at 3, 6, 9, and 12 months	QALY differences were not significant; overall cardiac health costs were somewhat non-significantly less in intervention despite significantly higher initial costs.
					I: telerehabilitation program with supported home training.			
Batalik et al., 2021 [36]	RCT (Czech Republic)	N=36	CBCR:57.1(7.9)	T2DM, HTN, dyslipidemia	Two groups receive home or center-based cardiac rehabilitation.	Peak Vo2, QoL, MACE hospitalizations and deaths.	Baseline, 12- and 15-months follow-up	Home-based training exhibited significantly higher peak Vo2 at year-end; and significant improvements in health quality in the general health area metrics and fitness. No difference in hospitalization rate between groups.
			HBCT:56.1(6.8)					
Bernal-Jiménez et al., 2024 [37]	RCT (Spain)	N=128	I: 57.70 (8.16)	T2DM, HTN, dyslipidemia, obesity	I: mHealth group with access to an app for monitoring	PA, QoL, MACE	Baseline and 9 months	mHealth users reported significantly more PA; significant improvements in the physical component of QoL among interventions; and other dimensions similar across groups.
			C: 61.46 (9.47)		C: received standard care without the app.			
Redfern et al., 2023 [38]	RCT (Australia)	N=115	C:64.3(8.9)	T2DM, HTN, dyslipidemia, previous MI, cardiomyopathy, peripheral vascular disease, stroke.	C: Usual care;	6-MWD, lifestyle, patient-reported outcome and experience measures, medication adherence, and rehabilitation attendance.	Baseline and 6 months	No significant differences in physical outcomes(6MWD), QOL, and METS; significantly lower scores for anxiety and depression in intervention vs control at 6 months.
			I:61.6(11.7)		I: text messaging program for lifestyle support.			
Avila et al., 2020 [39]	RCT (Belgium/UK)	N=90	HB:62.2(7.1)	HTN, T2DM	C: usual care	VO2 peak, Secondary: PA behavior, CV risk profile, and QoL	Outcome measures at 3 months	Maintained exercise capacity; all groups exhibited high QoL scores; but no significant differences were found.
			C:63.7(7.4)		I: Home-based telemonitoring exercise			
			CB:62.0(7.4)		Centre-based: supervised outpatient program.			
Su & Yu, 2021 [40]	RCT (China)	N=146	I:55.53(7.30)	CV comorbidities including hypertension, T2DM, and dyslipidemia.	I: mobile app usage for health tracking;	PA, health behaviors, smoking status, QoL, psychological well-being, cardiac physiological risk factors-	Baseline, 6 weeks, 12 weeks	Significant increases in steps/day were maintained post-intervention, as well as self-efficacy; and QoL improved significantly in the intervention group.
			C:56.03(7.02)		C: usual care with brief sessions about lifestyle changes.			
Widmer et al., 2017 [41]	RCT (USA)	N=80	C:63.6(10.9)	T2DM, HTN, dyslipidemia, metabolic syndrome	C: standard CR	ED visits, rehospitalizations, physiological outcomes, health behavior, QoL, stress, and smoking status	Baseline 30, 60 and 90 days	The intervention group reduced weight significantly and showed trends in lower rehospitalizations; no differences in PA or exercise capacity at 3 months.
			I:62.5(10.7)		I: DHI+CR program.			
Maddison et al., 2019 [42]	RCT (NZ)	N=162	Remote-CR: 61.0(13.2)	T2DM, HTN, hypercholesterolemia.	Remote-CR: 12 weeks of individualized exercise via a telerehabilitation platform.	VO2 max, CV risk factors, PA, HRQoL, MACE	VO2 max at 12 weeks; other metrics at 12 and 24 weeks	VO2 max comparable in both groups; lower program costs for remote CR, no significant differences in hospital service utilization.
			Centre-based CR: 61.5(12.2)		Centre-based: 12 weeks conventional CR.			
Snoek et al., 2021 [43]	RCT (Netherlands)	N=299	I:60.0(8.4)	T2DM, HTN smoking, Hypercholesterolemia	I: Telemonitoring and telecoaching	peakVO2, PA, QoL, rehospitalization, CV risk factors, MACE	6 months post-intervention measures conducted	Mean peak VO2 significantly improved in the intervention group, Habitual PA levels, QoL, and rehospitalization were comparable but No significant differences between groups.
			C:59.0(10.7)		C: Standard follow-up.			
Sankaran et al., 2019 [44]	Randomized crossover (Belgium)	N=28	Mean:60.9 (8.2)	T2DM, dyslipidemia, CAD, percutaneous coronary intervention, coronary artery bypass grafting, endoscopic atraumatic coronary artery bypass	Crossover design: Group 1 used the app first, followed by usual care; Group 2 is the reverse.	QoL, health parameters, exercise capacity, and risk factors	Baseline and 2 months	Significant carryover effects on exercise capacity; increase in QALY; self-reported health, anxiety reduction, and PA levels improved during app usage.
Frederix et al., 2016 [45]	RCT (Belgium)	N=338	I:61(9)	Atrial fibrillation, T2DM, hyperlipidemia, HTN	Both groups received 12-week CBCR	Healthcare costs, rehospitalizations, and QALYs gained.	12 and 24 weeks	The intervention group had significantly lower healthcare costs, number of days lost due to CV rehospitalizations, and improved QoL compared to controls; cost-effective as well.
			C:61(8)		Interventions included 24-week telerehabilitation alongside traditional methods.			
Frederix et al., 2015 [46]	RCT (Belgium)	N=140	I:61(9)	Atrial fibrillation, T2DM, hyperlipidemia, HTN	C: 12-week CBCR: 2x weekly sessions	Peak aerobic capacity, daily PA, QoL, and CV risk factors	6 weeks and 6 months	VO2 peak increased significantly in the intervention group; also, significant improvements in daily PA and HRQL scores were noted.
			C:61(8)		I: 24-week telerehabilitation starting week 6 with personalized protocols and telecoaching via SMS.			

Quality Assessment

The methodological quality of the included studies was assessed using the PEDro Scale. Eleven studies [34,36-38,40-46] were classified as good quality, while two others [35,39] were considered fair quality (Table 2) based on established cutoff scores [27,29]. The mean PEDro score of 6.69 across the 13 included studies indicates that the overall methodological quality of the included studies was good. None of the included studies were of poor quality. The two studies [35,39] were categorised as fair quality, but both studies [35,39] had significant methodological limitations. Lack of concealed allocation and baseline imbalances increased selection bias. The absence of blinding for participants, assessors, and therapists heightened the risk of performance and detection bias. Furthermore, study [39] lacked an intention-to-treat analysis.

**Table 2:** Methodological quality rating of all the selected studies based on the PEDro scale PEDro scale: Physiotherapy evidence database scale

Criterion	Herring et al., 2021 [34]	Brouwers, et al., 2021 [35]	Batalik et al., 2021 [36]	Bernal-Jiménez et al., 2024 [37]	Redfern et al., 2024 [38]	Avila et al., 2020 [39]	Su and Yu, 2021 [40]	Widmer et al., 2017 [41]	Maddison et al., 2019 [42]	Snoek et al., 2019 [43]	Sankaran et al., 2019 [44]	Frederix et al., 2016 [45]	Frederix et al., 2015 [46]
Eligibility criteria were specified	Yes	Yes	No	Yes	Yes	Yes	Yes	Yes	Yes	Yes	Yes	Yes	Yes
Subjects were randomly allocated to groups (in a crossover study, subjects were randomly allocated an order in which treatments were received)	Yes	Yes	Yes	Yes	Yes	Yes	Yes	Yes	Yes	Yes	Yes	Yes	Yes
Allocation was concealed	Yes	No	No	No	Yes	No	No	No	Yes	No	No	No	No
The groups were similar at baseline regarding the most important prognostic indicators	Yes	No	Yes	Yes	Yes	Yes	Yes	Yes	Yes	Yes	Yes	Yes	Yes
There was blinding of all subjects	No	No	No	No	No	No	No	No	No	No	No	No	No
There was blinding of all therapists who administered the therapy	No	No	No	No	No	No	No	No	No	No	No	No	No
There was blinding of all assessors who measured at least one key outcome	Yes	No	No	Ye	Yes	No	Yes	Yes	Yes	No	Yes	Yes	Yes
Measures of at least one key outcome were obtained from more than 85% of the subjects initially allocated to groups	Yes	Yes	Yes	Yes	Yes	Yes	Yes	Yes	Yes	Yes	Yes	Yes	Yes
All subjects for whom outcome measures were available received the treatment or control condition as allocated or, where this was not the case, data for at least one key outcome was analysed by “intention to treat”	Yes	Yes	Yes	Yes	Yes	No	Yes	No	Yes	Yes	Yes	Yes	Yes
The results of between-group statistical comparisons are reported for at least one key outcome	Yes	Yes	Yes	Yes	Yes	Yes	Yes	Yes	Yes	Yes	Yes	Yes	Yes
The study provides both point measures and measures of variability for at least one key outcome	Yes	Yes	Yes	Yes	Yes	Yes	Yes	Yes	Yes	Yes	Yes	Yes	Yes
Total points (out of 10)	8	5	6	7	8	5	7	6	8	6	7	7	7

Risk of Bias in Included Studies

**Figure 2:**
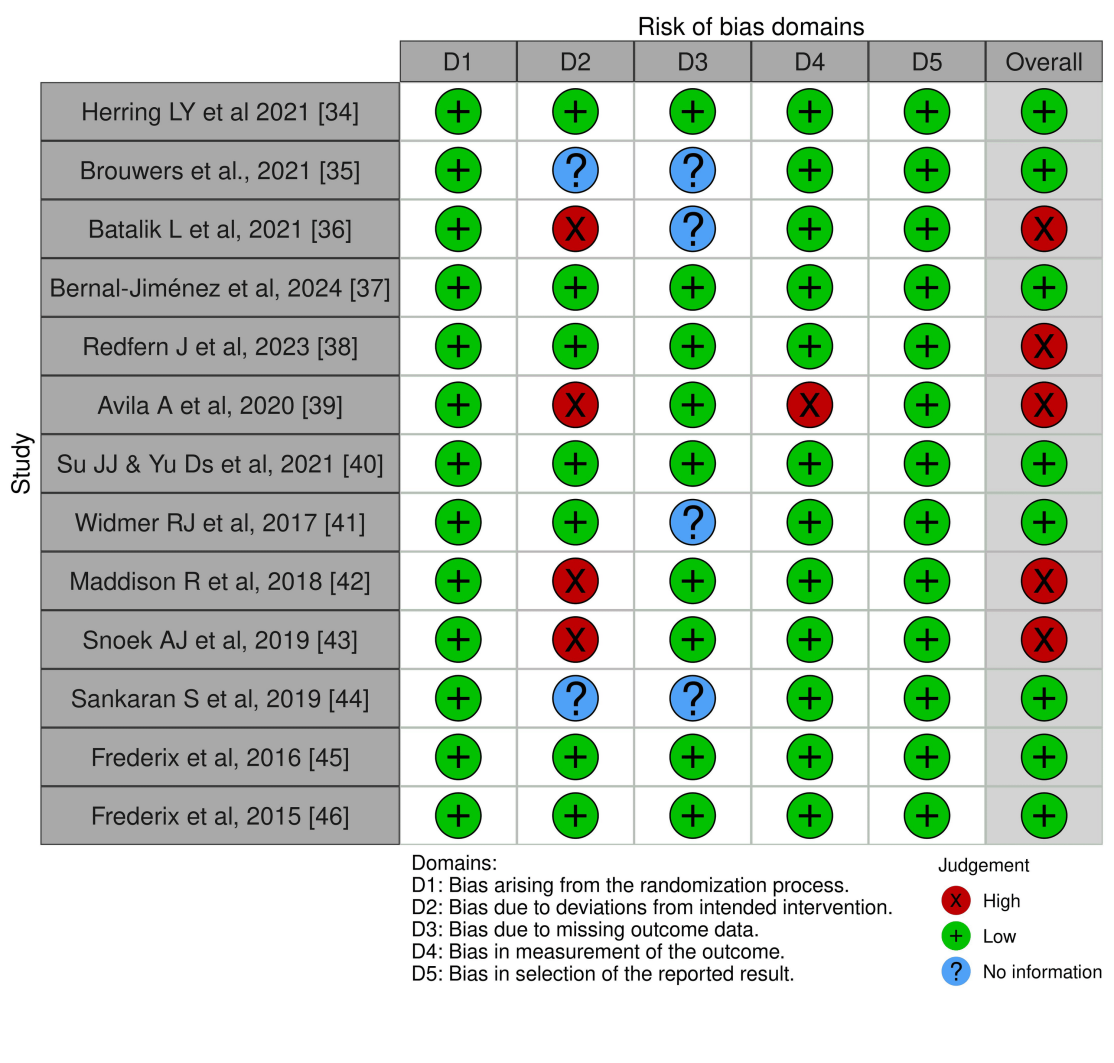
Cochrane risk-of-bias tool for randomized trials (RoB 2)

**Figure 3:**
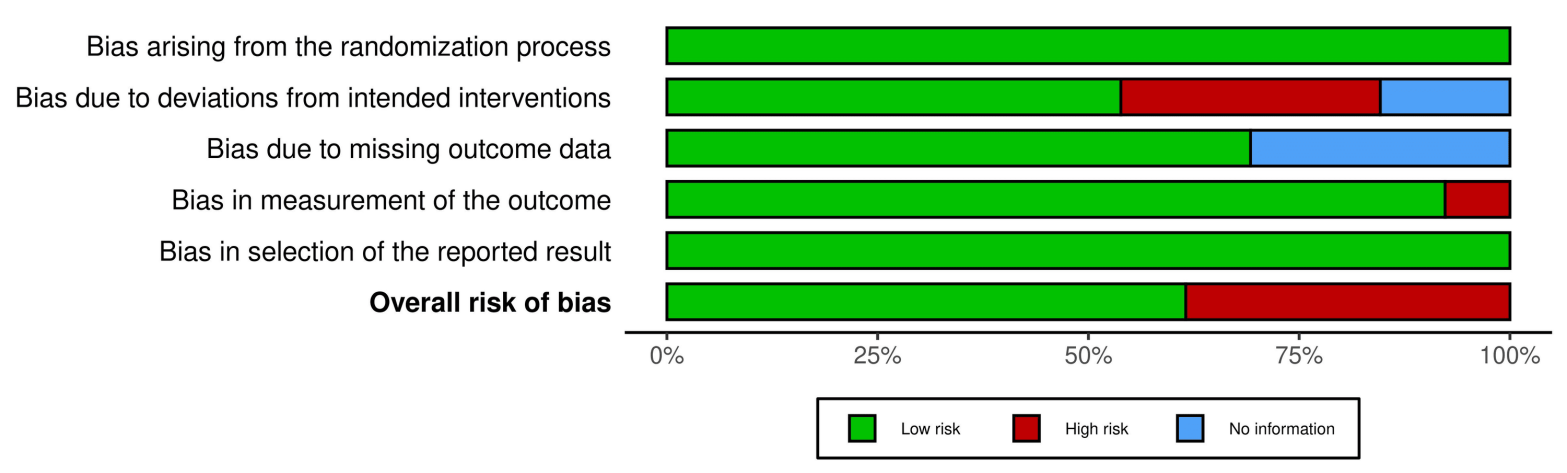
Summary plot for risk of bias assessment for the included studies

The quality of the included studies was evaluated using the Cochrane Risk of Bias tool. All 13 studies [34-46] demonstrated a low risk of bias related to randomisation. Seven RCTs [34,37,38,40,41,45,46] implemented blinding of either participants or personnel, thereby minimising performance bias. However, five studies [35,36,39,42,43] exhibited a high risk of bias, and one study [44] presented an unclear risk. Furthermore, eight studies [34,35,37,40,42,43,45,46] employed blinding for outcome assessors, which helped reduce bias, whereas the remaining five studies [36,38,39,41,44] did not report this information, resulting in an unclear risk of bias. Instances of incomplete outcome data were minimal, with 11 studies [34,35,37,38,40-46] categorised as having a low risk of bias, one study [36] classified as having an unclear risk, and one study [39] as high risk in this domain. There was no evidence of selective reporting bias, as all studies reported outcomes at all specified time points, and all studies utilised appropriate statistical methods. ​Overall, although the majority of the studies exhibited a low risk of bias, methodological quality varied among the included studies (Figures 2, 3).

Interventions and Outcome Measures

Studies have demonstrated a range of interventions aimed at enhancing the outcomes of patients with CR (Table 1). Herring et al. (2021) utilised a combination of a British Heart Foundation (BHF) leaflet, PACES education delivered in two sessions of 2.5 hours each, and motivational text messages in their intervention group as compared to general health advice for the control group [34]. In the study conducted by Frederix et al. (2015), participants underwent a 12-week centre-based CR program with two sessions weekly, followed by 24 weeks of telerehabilitation starting in week six, which included personalised protocols and telecoaching via short messaging service (SMS) [46]. A similar approach was implemented by Frederix et al. (2016), where both groups received a 12-week centre-based rehabilitation, but the intervention involved an additional 24 weeks of telerehabilitation [45]. Maddison et al. (2019) implemented the REMOTE-CR program, which offered individualized exercise through a telerehabilitation platform alongside conventional center-based CR [42]. Snoek et al. (2021) used telemonitoring and telecoaching as an intervention contrasted with standard follow-up care in their study [43]. In a randomised crossover design employed by Sankaran et al. (2019), participants first utilised a mobile app before transitioning to usual care, whereas the opposing group followed the reverse order [44]. Avila et al. (2020) contrasted home-based telemonitoring exercises with traditional supervised outpatient programs as control [39]. Widmer et al. (2017) assessed a digital health intervention combined with a standard CR programme [41]. Su and Yu (2021) examined the influence of mobile app usage for health tracking against a control that received brief lifestyle change sessions [40]. Bernal et al. focused on a mHealth group utilising a monitoring application compared to a control group without app usage [37]. Redfern et al. (2023) [38] evaluated a text messaging program for lifestyle support against usual care, whereas Batalik et al. [36] investigated the effects of home-based versus centre-based CR. Brouwers et al. studied a telerehabilitation program with supported home training compared with group-based exercise [35]. Collectively, these interventions demonstrate the evolving landscape of CR.

The interventions examined in the studies displayed considerable variability in duration.​ One study [34] implemented two sessions, each lasting 2.5 hours, approximately two weeks apart, followed by text message support (Table 1). Alternatively, two studies reported a three-month intervention duration [39,41]. Additionally, four studies employed interventions lasting four months [36,40,42,44]. In contrast, two studies implemented interventions over six months [38,43]. Furthermore, two studies [37,45] had interventions lasting nine months, while a single study [35] utilized a twelve-month intervention duration (Table 1). The mean duration of the interventions across all included studies was found to be 5.3 months. Several of the included studies performed follow-up assessments, with the duration of follow-up varying among them.​ Specifically, seven studies [34-36,39,43,45] utilised a follow-up period of one year, whereas one study [38] was conducted with a six-month follow-up duration. Additionally, one study implemented a follow-up period of four months (Table 1) [42]. The variable duration of follow-up can influence the study findings as the shorter follow-up duration studies may not have captured the longer-term effects, whereas the longer duration follow-up studies could provide an insight into the sustainability of the rehab programs. DCR showed higher economic and healthcare benefits over traditional rehab in the longer-duration studies, although these effects were more pronounced in the shorter-duration follow-up studies.

The present systematic review and meta-analysis examined several key variables of interest across various studies.​ Physical activity was one of the primary variables, with relevant findings reported in various studies [34,37-42,44,46]. Another significant focus was QoL [34-44,46]. Additionally, the review explored rehospitalization rates [35,36,41,45], costs [35,45], emergency department visits [41], and occurrence of MACE [34,36-38,41-43] (Table 1).

Other outcome measures assessed in the included studies were haemoglobin (Hb)A1c [34,46], peak aerobic capacity (VO2 peak) [36,39,43,46], glycemic control [39,46], lipid profile [34,39,41,42,43,46], differential incremental quality-adjusted life years (QALYs) gained [45], VO2 max [42], glucose concentrations [41,42], anthropometric data [39,42], blood pressure (systolic/diastolic) [42], exercise-related motivation [42], exercise adherence [42], utilization of care [43], deaths [36], qualitative data on users’ motivation, user experience, exercise capacity [44], muscle function [39], health behaviors [40], cardiac self-efficacy [40], psychological wellbeing [37,40] cardiac physiological risk factors [37,40], six-minute walk distance [38], anxiety and depression [38].

Results of meta-analysis

A meta-analysis was performed on four variables: QoL, physical activity levels, rehospitalisations, and MACE.

Quality of Life

**Figure 4:**
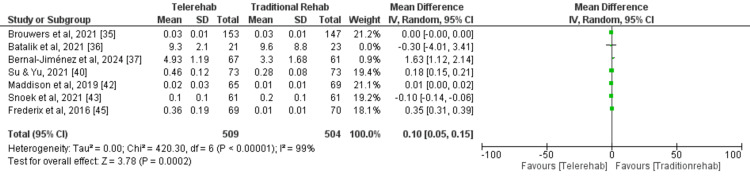
A forest plot for quality of life for studies included in the meta-analysis

A meta-analysis (random effects model) of seven studies [35-37,40,42,43,45] was conducted to evaluate the impact of DCR on QoL compared with traditional rehabilitation. A total of 509 and 504 participants were enrolled in the digital and traditional rehabilitation groups, respectively. The pooled analysis revealed a statistically significant improvement in outcomes with digital rehabilitation compared with traditional rehabilitation (MD = 0.10, 95% CI: 0.05-0.15, p = 0.0002) (Figure 4).

**Figure 5:**
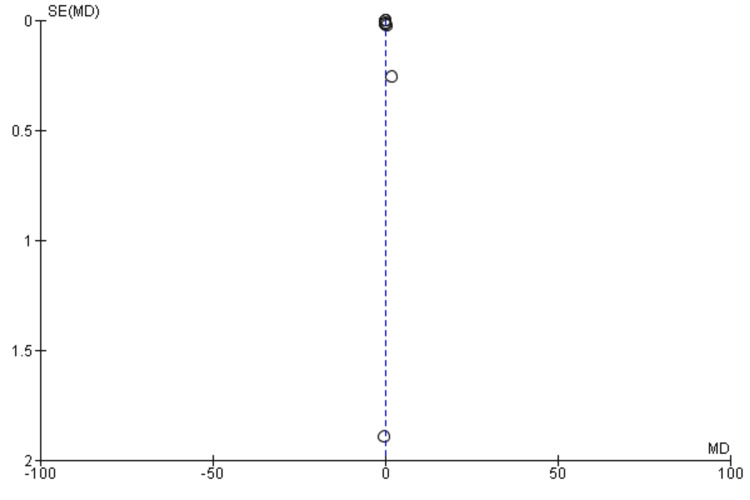
A funnel plot demonstrating no obvious publication bias for studies included in the quality of life analysis

This finding suggests that digital rehabilitation may be more effective than traditional rehabilitation. High heterogeneity was observed among the included studies (I2 = 99%, p < 0.00001), indicating inconsistent results across studies, however the sensitivity analysis did not show any difference by using either random or fixed effect models or exclusion of studies. The high heterogeneity is due to multiple factors, including follow-up duration, sample size and outcome measurement. The funnel plot did not demonstrate any potential publication bias (Figure 5).

Physical Activity Levels

**Figure 6:**
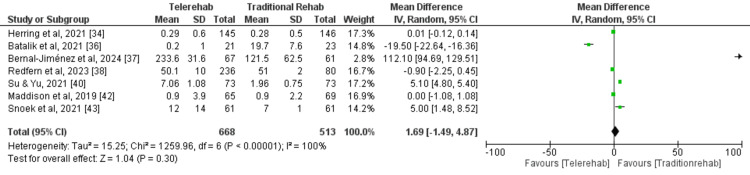
A forest plot for physical activity for studies included in the meta-analysis

**Figure 7:**
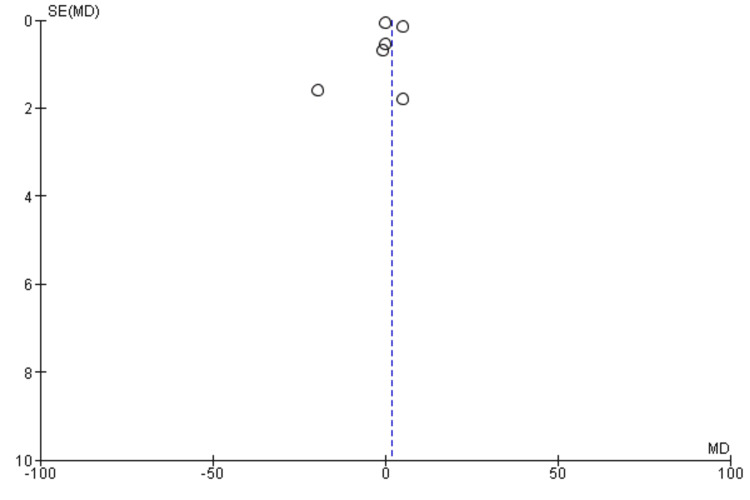
A funnel plot demonstrating no obvious publication bias

A meta-analysis (random effects model) of seven studies [34,36-38,40,42,43] was conducted to evaluate the impact of DCR on physical activity levels compared with traditional rehabilitation. A total of 668 and 513 participants were enrolled in the digital and traditional rehabilitation groups, respectively. The pooled analysis revealed a statistically non-significant improvement in physical activity levels with digital rehabilitation compared to traditional rehabilitation (MD= 1.69, 95% CI: 1.49-4.87, p = 0.30) (Figure 6). This suggests that digital rehabilitation may not be more effective than traditional rehabilitation. High heterogeneity was observed among the included studies (I2 = 100%, p < 0.00001), indicating inconsistent results across studies. The funnel plot did not demonstrate any potential publication bias (Figure 7). 

Rehospitalization

**Figure 8:**
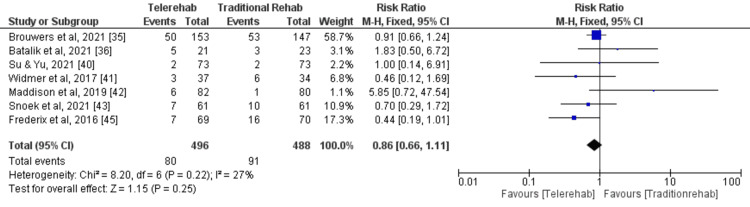
A forest plot for rehospitalisation for patients included in the meta-analysis

A meta-analysis (fixed effects model) of seven studies [35,36,40-43,45] was conducted to evaluate the impact of DCR on rehospitalisation compared with traditional rehabilitation. A total of 496 and 488 participants were enrolled in the digital and traditional rehabilitation groups, respectively. The pooled analysis revealed a statistically non-significant reduction in the risk of rehospitalisation with digital rehabilitation compared with traditional rehabilitation (RR = 0.86, 95% CI: 0.66-1.11, p = 0.25) (Figure 8).

**Figure 9:**
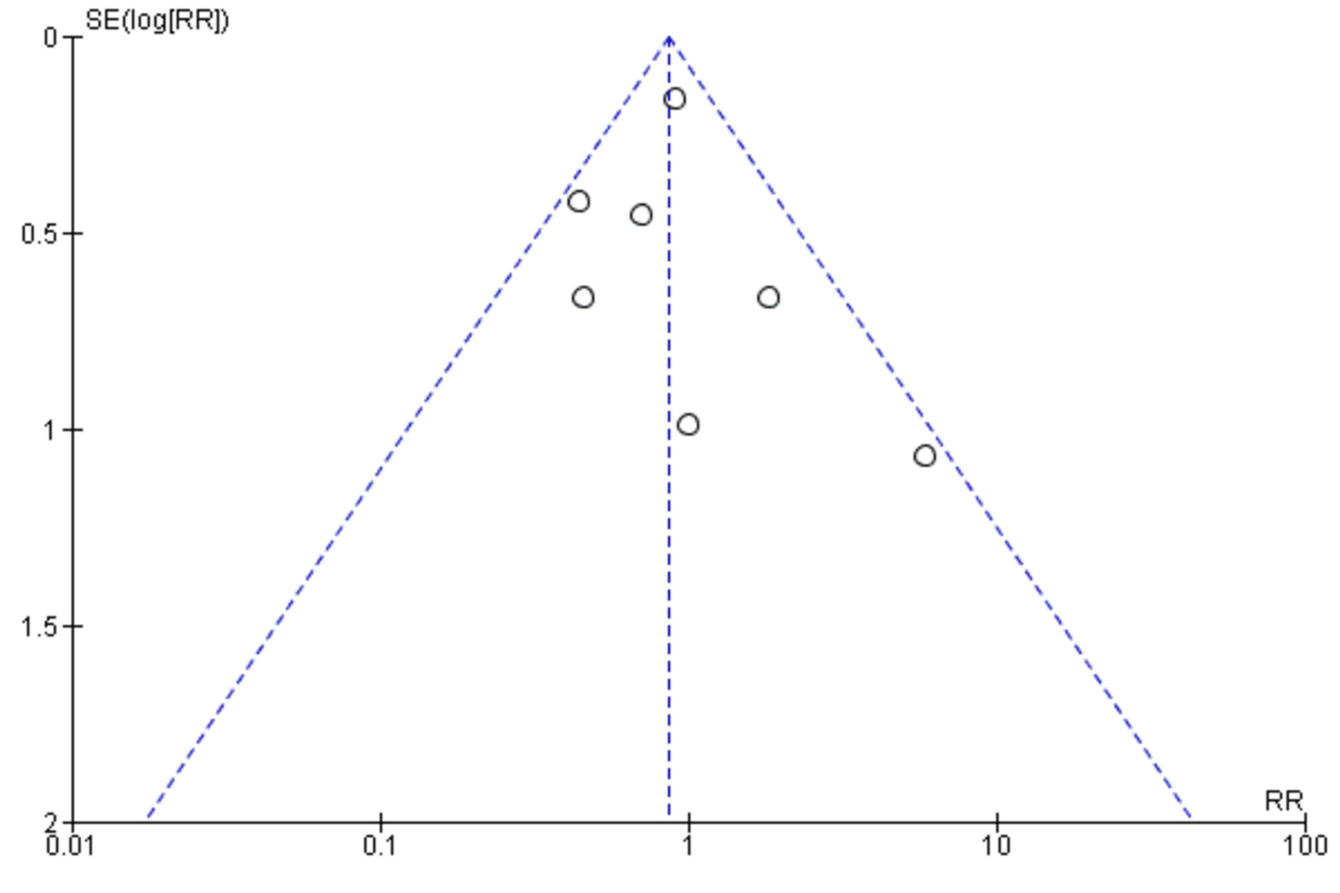
A funnel plot demonstrating no obvious publication bias

This suggests that digital rehabilitation may be associated with a lower risk of adverse events; however, the evidence is not conclusive. No significant heterogeneity was observed among the included studies (I2 = 27%, p = 0.22), indicating that the results were consistent across studies. The funnel plot did not demonstrate any potential publication bias (Figure 9).

Major Adverse Cardiovascular Events

**Figure 10:**
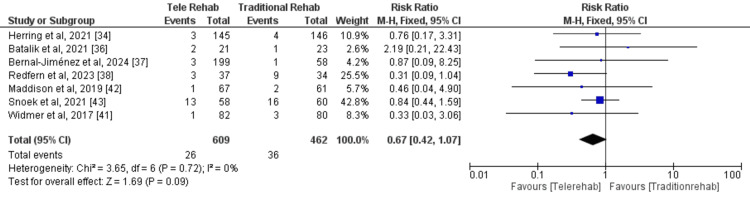
Major adverse cardiovascular events in the participants

A meta-analysis (fixed effects model) of seven studies [34,36-38,41-43] was conducted to evaluate the impact of DCR on rehospitalisation compared with traditional rehabilitation. A total of 609 and 462 participants were enrolled in the digital and traditional rehabilitation groups, respectively. The pooled analysis revealed a statistically non-significant reduction in the risk of adverse cardiovascular events with digital rehabilitation compared with traditional rehabilitation (RR = 0.67, 95% CI: 0.42-1.07, p = 0.09) (Figure 10).

**Figure 11:**
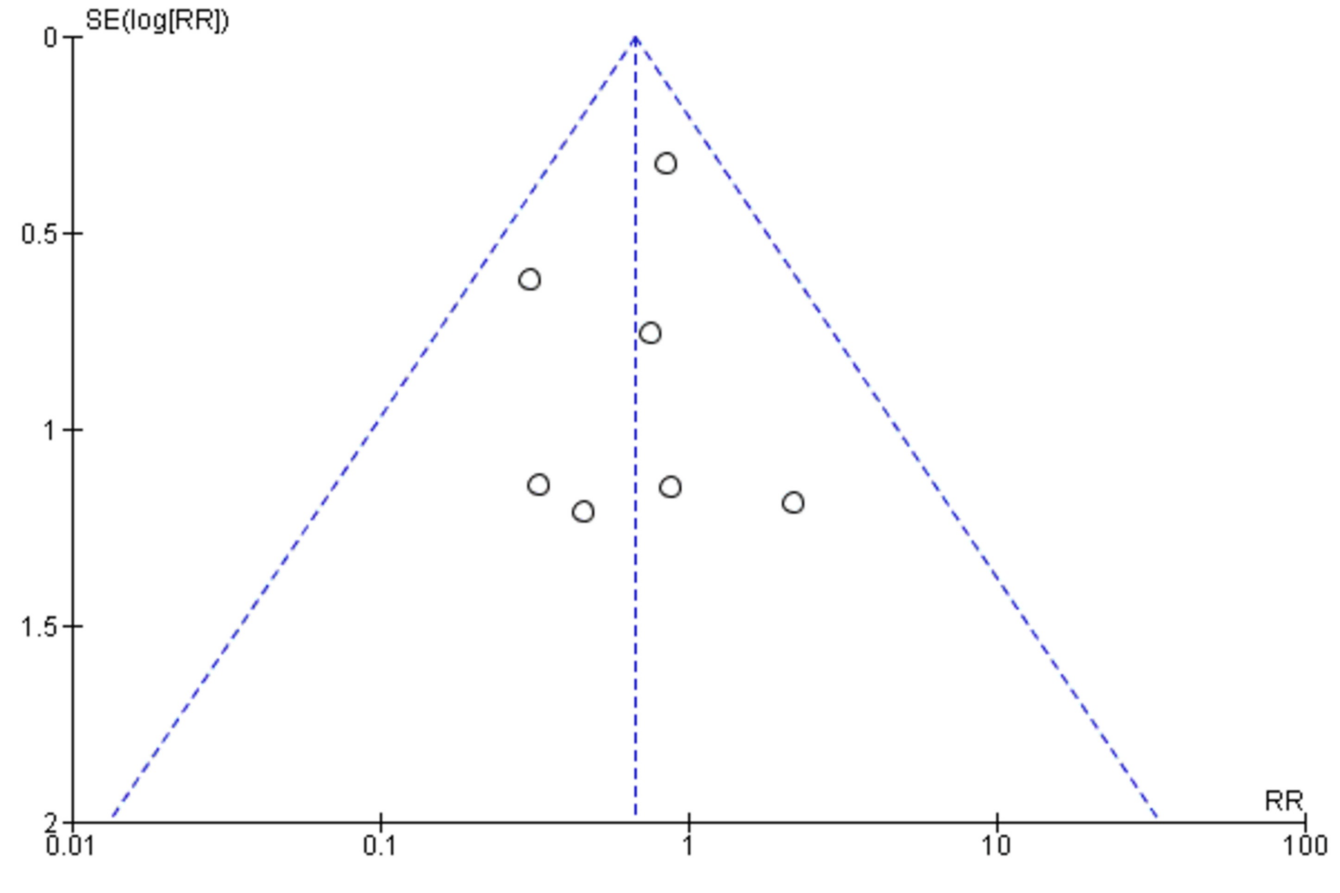
The funnel plot for risk of publication for the studies included in this meta-analysis

This suggests that digital rehabilitation may be associated with a lower risk of adverse events, but the evidence is not conclusive. No significant heterogeneity was observed among the included studies (I2 = 0%, p = 0.72), indicating that the results were consistent across studies. The funnel plot did not demonstrate any potential publication bias (Figure 11).

Costs

**Figure 12:**
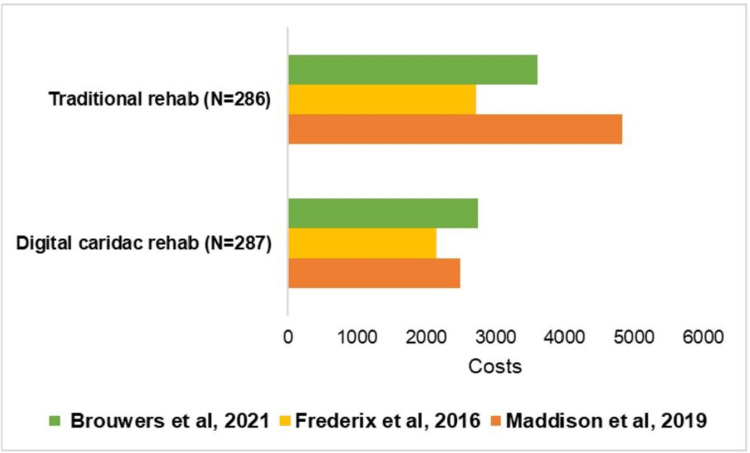
The number of patients and total costs per patients enrolled in the traditional and digital cardiac rehab programs Brouwer et al, 2021 [35], Frederix et al, 2016 [45], Maddison et al, 2019 [42].

Data regarding costs were provided in three studies [35,42,45]. Because a meta-analysis was not feasible with this limited number of studies, the collective data from these studies are represented in a bar graph (Figure 12). There were 287 and 286 participants in the DCR and traditional rehabilitation groups, respectively.

Sensitivity and subgroup analysis for MACE

**Figure 13:**
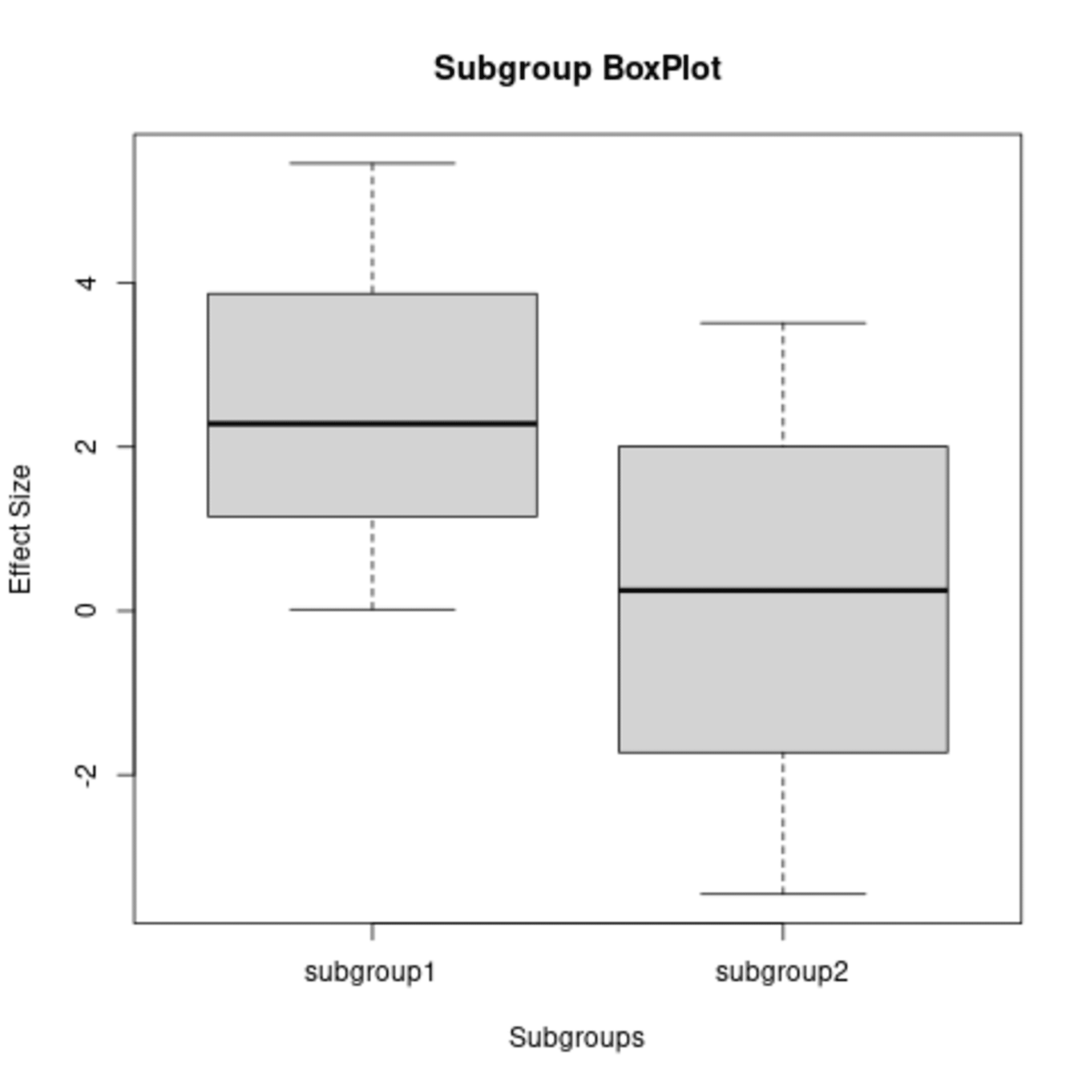
Box plot for the studies based on distribution into low- and high-risk categories Subgroup 1 and subgroup 2 show studies with low risk and high risk of bias respectively.

The subgroup analysis is based on the distribution of studies into high- and low-risk categories based on risk of bias assessment (Figure 13). Subgroups 1 and 2 include studies with low risk and high risk of bias, respectively. Subgroup 1 plot shows better agreement between the studies compared to subgroup 2. The plots also show the difference of opinion about the effectiveness of telerehabilitation versus traditional rehab. Similarly, the median values for the two groups are also different.

**Figure 14:**
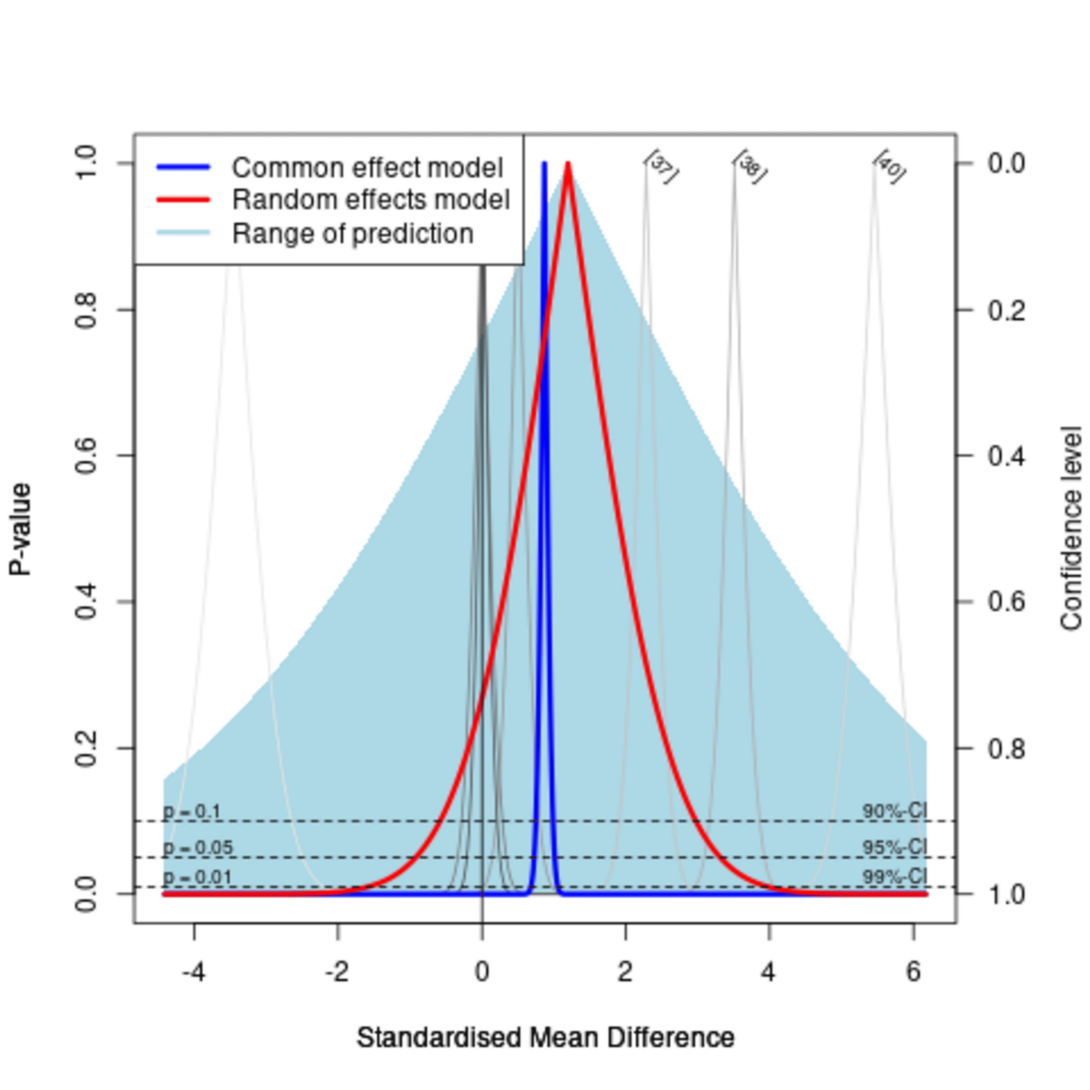
The Drapery plot for the included studies in the meta-analysis

The Drapery plot also shows significant heterogeneity between the studies (Figure 14). The gray curves correspond to primary studies, with study weights from the random effects model represented on a greyscale, with dark grey showing studies with higher precision and light grey showing studies with low precision, respectively. The light blue or prediction region is broader than the P-value curve of the pooled estimates (red and black curves), indicating heterogeneity. The sensitivity analysis did not show any major difference in the heterogeneity based on the exclusion of one study at a time.

Discussion

The present systematic review and meta-analysis aimed to evaluate the impact of DCR on significant clinical outcomes such as QoL, physical activity levels, hospital readmissions, MACE, and costs in patients with CAD. These findings suggest that DCR can enhance health outcomes, particularly QoL, while presenting mixed results regarding hospital readmissions and MACE.

This systematic review included 12 RCTs and one randomised crossover trial encompassing a diverse population of 1,850 individuals. The substantial male dominance within the studies reflects a notable trend in CAD research, which may influence how the results are generalised to a wider population, including a significant proportion of women. This gender disparity is consistent with previous findings [47] and women do not participate in CR to the same degree as men [48]. This disparity stems from various factors, including lower referral rates, lower enrollment, and lower adherence among women [49]. Potential reasons include lack of provider endorsement, transportation and childcare challenges, and exercise preferences that are often unmet (e.g., preference for yoga and dance over traditional equipment) [50,51]. To address the specific challenges women face in accessing and benefiting from CR, "women-focused" interventions can be developed. These interventions may involve adapting program content to women's unique needs and preferences, often referred to as "gender-tailored" approaches. The methodological quality of the included studies was high, with an average PEDro score of 6.69, indicating good methodological rigor. The use of the Cochrane Risk of Bias tool further supported the reliability of the findings, as most studies displayed a low risk of bias related to randomisation and blinding processes. However, some studies' lack of detailed reporting on blinding for outcome assessments and the observed variability in methodological quality across studies highlight potential limitations within the literature.

The meta-analysis revealed statistically significant improvements in QoL with DCR compared to traditional rehabilitation. These findings suggest that DCR can effectively enhance patient outcomes in this domain. However, high heterogeneity was observed for this measure (I2 = 99%), indicating considerable variability across studies that warrants careful interpretation. Several studies have confirmed the equivalence of telerehabilitation and centre-based rehabilitation. Previous studies [47,52-57] have demonstrated that digital telerehabilitation is either equally effective or superior to CBR in terms of adverse event rates, readmission rates, physical activity levels, adherence to physical activity guidelines, and lipid profiles. DCR is clinically equivalent to standard rehabilitation while offering advantages in patient adherence, motivation, and the promotion of sustained behavioural modifications [47,52,54]. While the meta-analysis on rehospitalisations and MACE revealed a trend towards reduced rehospitalisation rates and MACE with DCR, these findings did not reach statistical significance. In contrast, a previous study revealed that mobile-health-enhanced disease management programs significantly reduced the rates of overall, cardiac-related, and emergency department events compared with traditional disease management programs [58]. The findings of the present study could be attributed to several factors, including the relatively small number of studies included in the analysis. Although the cost analysis was limited by the small number of studies reporting this outcome, the available data suggest potential cost advantages of DCR. However, the initial investment in technological infrastructure and resources required for program customisation must be considered. Future research should incorporate comprehensive economic analyses to better understand the long-term cost implications of implementing DCR.

The analysis of DCR implementation revealed several important considerations. While DCR offers advantages in terms of accessibility and convenience, its successful deployment requires adequate technological infrastructure and patient capability. The effectiveness of DCR programs varies considerably in duration, ranging from brief interventions to year-long programs. This variability suggests the need for standardisation in program delivery while maintaining flexibility to meet individual patient needs. Telerehabilitation, like any other technology, presents both advantages and challenges [53]. While it offers cost-effectiveness, particularly in monitoring and evaluation, and increased accessibility for patients with limited mobility, it also presents potential drawbacks. These include the loss of direct patient-provider interaction and the resource-intensive nature of personalising programs. Furthermore, certain patient populations, such as the elderly with cognitive impairment, may face difficulties in effectively utilising technology [58]. Successful implementation relies heavily on robust technical infrastructure, including reliable Internet and strong mobile network coverage. The absence of direct physical contact can limit the scope of clinical assessments and potentially affect the overall effectiveness of the intervention.

This review has several important clinical implications. DCR offers a convenient and accessible alternative to traditional CR, potentially increasing patient participation and improving long-term outcomes. Healthcare providers should consider incorporating DCR into their care plans for patients with CAD, particularly those who face barriers to accessing traditional programs. DCR offers significant advantages for patients with comorbidities, elderly individuals, and those with mobility issues who may face challenges attending traditional CR programs. By eliminating the need for travel and offering flexibility, DCR improves accessibility for these populations. To further enhance equitable access, healthcare systems can implement strategies such as subsidizing program costs, partnering with community organizations to provide technology and support, and offering hybrid models that combine in-person and digital components. Successful DCR implementation requires integrating DCR platforms with electronic health records, providing comprehensive training for healthcare providers, and developing clear clinical pathways. Furthermore, ensuring reliable internet connectivity, providing dedicated technical support, and conducting ongoing evaluations are crucial for the long-term success and sustainability of DCR programs.

Study limitations

The present study has some limitations in that the variability in intervention types, durations, and outcome measures complicates direct comparisons across studies. Some of the limitations of the present study were the heterogeneity and lack of details provided in the articles analysed. The exclusion of non-English studies may have introduced publication bias and limited the generalizability of findings. Studies conducted in other languages may have different methodologies, populations, and outcomes, potentially presenting a more thorough understanding of the effectiveness of DCR. The review highlights the heterogeneity in DCR interventions, including significant variability in program durations. This variability can confound the interpretation of results, as shorter programs may have different results compared to longer, more intensive programs. Future research should investigate the optimal duration and intensity of DCR programs for different patient populations. These limitations have been acknowledged and discussed in greater detail in the revised manuscript to provide a more comprehensive and nuanced understanding of the strengths and weaknesses of the current body of evidence.

The predominance of male participants (79.3%) in the included studies limited the generalisability to the broader CAD population. Additionally, most telerehabilitation interventions select only physical activity and risk factor management. Further research on telerehabilitation that integrates all core components in one intervention thus seems highly desirable. In future RCTs, it is essential to include more diverse patient populations to enhance the generalizability of findings across different groups with CVD. Standardizing intervention protocols across studies is also crucial to ensure comparability and consistency in the delivery of DCR interventions. Furthermore, incorporating other important outcomes such as psychological status, nutrition, social interaction, and cost-effectiveness analyses in RCTs is necessary to assess the viability of DCR programs relative to traditional rehabilitation models. These steps will help address key gaps in the current evidence base and provide a more comprehensive understanding of the potential benefits and limitations of DCR interventions.

## Conclusions

This systematic review and meta-analysis found that DCR improves QoL in patients with CAD compared with traditional rehabilitation. While trends towards reduced rehospitalisations and MACE were observed, these did not reach statistical significance.. However, it is important to interpret the observed improvements in QoL with caution, as definitive causality cannot be established. The non-significant results for rehospitalizations and MACE highlight the uncertainty surrounding these findings, underscoring the need for more robust evidence to confirm these trends conclusively. DCR offers advantages such as improved patient adherence and convenience; however, challenges such as technology access and limited personal interaction need to be addressed. As technology continues to evolve and healthcare systems adapt to changing patient needs, DCR is likely to play an increasingly important role in the comprehensive delivery of cardiovascular care. For future research, it is suggested that studies examine the long-term sustainability of DCR benefits, particularly over one year or more, and explore the impact of DCR on specific subgroups, such as elderly patients or those with multiple comorbidities. Incorporating economic evaluations and patient-reported outcomes (PROs) in RCTs is also recommended to assess the cost-effectiveness and patient-centered outcomes of DCR intervention. Future research should focus on larger-scale studies and optimise DCR programs to maximise patient engagement and improve clinical outcomes. Strategies to optimize DCR may include the use of personalized care plans tailored to individual patient needs and preferences, which can foster a sense of ownership and accountability. Additionally, gamification techniques, such as setting achievable goals, providing rewards, and integrating interactive challenges to enhance patient engagement, can be used. These approaches, combined with regular feedback and support from healthcare providers, can create a more engaging and sustainable experience for patients. To address challenges in technology access, strategies such as developing more user-friendly digital platforms, offering patient training, and exploring hybrid models combining in-person and digital care should be considered. These approaches will help overcome barriers and enhance the effectiveness and accessibility of the DCR program.

## Appendices

**Table 3:** Search keywords utilized for literature search

Scopus
"Coronary artery disease" OR "CAD" AND "Digital cardiac rehabilitation" OR "Digital rehabilitation" OR "Digital rehab" OR "Telerehabilitation" OR "Telerehab" OR " Virtual rehabilitation" AND "Hospital readmissions" OR "Costs" OR "Physical activity" OR "Physical activity levels" OR "Qulaity of life” OR "Major adverse cardiovascular events”
Web of Science
"Coronary artery disease" OR "CAD" AND "Digital cardiac rehabilitation" OR "Digital rehabilitation" OR "Digital rehab" OR "Telerehabilitation" OR "Telerehab" OR " Virtual rehabilitation" AND "Hospital readmissions" OR "Costs" OR "Physical activity" OR "Physical activity levels" OR "Qulaity of life” OR "Major adverse cardiovascular events”
Pubmed
"Coronary artery disease" OR "CAD" AND "Digital cardiac rehabilitation" OR "Digital rehabilitation" OR "Digital rehab" OR "Telerehabilitation" OR "Telerehab" OR " Virtual rehabilitation" AND "Hospital readmissions" OR "Costs" OR "Physical activity" OR "Physical activity levels" OR "Qulaity of life” OR "Major adverse cardiovascular events”

## References

[1] Kang EH, Park EH, Shin A, Song JS, Kim SC (2021). Cardiovascular risk associated with allopurinol vs. benzbromarone in patients with gout. Eur Heart J.

[2] Christensen RH, Wedell-Neergaard AS, Lehrskov LL (2019). Effect of aerobic and resistance exercise on cardiac adipose tissues: secondary analyses from a randomized clinical trial. JAMA Cardiol.

[3] Choi S, Kim K, Kim SM (2018). Association of obesity or weight change with coronary heart disease among young adults in South Korea. JAMA Intern Med.

[4] Malakar AK, Choudhury D, Halder B, Paul P, Uddin A, Chakraborty S (2019). A review on coronary artery disease, its risk factors, and therapeutics. J Cell Physiol.

[5] Anderson L, Thompson DR, Oldridge N, Zwisler AD, Rees K, Martin N, Taylor RS (2016). Exercise-based cardiac rehabilitation for coronary heart disease. Cochrane Database Syst Rev.

[6] Knuuti J, Wijns W, Saraste A (2020). 2019 ESC Guidelines for the diagnosis and management of chronic coronary syndromes. Eur Heart J.

[7] Anderson L, Oldridge N, Thompson DR, Zwisler AD, Rees K, Martin N, Taylor RS (2016). Exercise-based cardiac rehabilitation for coronary heart disease: cochrane systematic review and meta-analysis. J Am Coll Cardiol.

[8] Eijsvogels TM, Maessen MF, Bakker EA (2020). Association of cardiac rehabilitation with all-cause mortality among patients with cardiovascular disease in the Netherlands. JAMA Netw Open.

[9] De Gruyter E, Ford G, Stavreski B (2016). Economic and social impact of increasing uptake of cardiac rehabilitation services--a cost benefit analysis. Heart Lung Circ.

[10] Kotseva K, Wood D, De Bacquer D (2018). Determinants of participation and risk factor control according to attendance in cardiac rehabilitation programmes in coronary patients in Europe: EUROASPIRE IV survey. Eur J Prev Cardiol.

[11] Rawstorn JC, Gant N, Direito A, Beckmann C, Maddison R (2016). Telehealth exercise-based cardiac rehabilitation: a systematic review and meta-analysis. Heart.

[12] Scherrenberg M, Falter M, Dendale P (2020). Cost-effectiveness of cardiac telerehabilitation in coronary artery disease and heart failure patients: systematic review of randomized controlled trials. Eur Heart J Digit Health.

[13] Subedi N, Rawstorn JC, Gao L, Koorts H, Maddison R (2020). Implementation of telerehabilitation interventions for the self-management of cardiovascular disease: systematic review. JMIR Mhealth Uhealth.

[14] Huang K, Liu W, He D (2015). Telehealth interventions versus center-based cardiac rehabilitation of coronary artery disease: a systematic review and meta-analysis. Eur J Prev Cardiol.

[15] Janssen V, De Gucht V, van Exel H, Maes S (2013). Beyond resolutions? A randomized controlled trial of a self-regulation lifestyle programme for post-cardiac rehabilitation patients. Eur J Prev Cardiol.

[16] Tekkeşin Aİ, Hayıroğlu Mİ, Çinier G (2021). Lifestyle intervention using mobile technology and smart devices in patients with high cardiovascular risk: a pragmatic randomised clinical trial. Atherosclerosis.

[17] Hayıroğlu Mİ, Çınar T, Çinier G (2021). The effect of 1-year mean step count on the change in the atherosclerotic cardiovascular disease risk calculation in patients with high cardiovascular risk: a sub-study of the LIGHT randomized clinical trial. Kardiol Pol.

[18] Hayıroğlu Mİ, Çınar T, Cilli Hayıroğlu S, Şaylık F, Uzun M, Tekkeşin Aİ (2023). The role of smart devices and mobile application on the change in peak VO(2) in patients with high cardiovascular risk: a sub-study of the LIGHT randomised clinical trial. Acta Cardiol.

[19] Widmer RJ, Collins NM, Collins CS, West CP, Lerman LO, Lerman A (2015). Digital health interventions for the prevention of cardiovascular disease: a systematic review and meta-analysis. Mayo Clin Proc.

[20] Widmer RJ, Allison TG, Lerman LO, Lerman A (2015). Digital health intervention as an adjunct to cardiac rehabilitation reduces cardiovascular risk factors and rehospitalizations. J Cardiovasc Transl Res.

[21] Shields GE, Wells A, Doherty P, Heagerty A, Buck D, Davies LM (2018). Cost-effectiveness of cardiac rehabilitation: a systematic review. Heart.

[22] Frederix I, Vanhees L, Dendale P, Goetschalckx K (2015). A review of telerehabilitation for cardiac patients. J Telemed Telecare.

[23] Jin K, Khonsari S, Gallagher R (2019). Telehealth interventions for the secondary prevention of coronary heart disease: a systematic review and meta-analysis. Eur J Cardiovasc Nurs.

[24] Cruz-Cobo C, Bernal-Jiménez MÁ, Vázquez-García R, Santi-Cano MJ (2022). Effectiveness of mHealth interventions in the control of lifestyle and cardiovascular risk factors in patients after a coronary event: systematic review and meta-analysis. JMIR Mhealth Uhealth.

[25] Page MJ, McKenzie JE, Bossuyt PM (2021). The PRISMA 2020 statement: an updated guideline for reporting systematic reviews. BMJ.

[26] McKenzie JE, Brennan SE, Ryan RE, Thomson HJ, Johnston RV, Thomas J Defining the criteria for including studies and how they will be grouped for the synthesis. Cochrane handbook for systematic reviews of interventions.

[27] Foley NC, Bhogal SK, Teasell RW, Bureau Y, Speechley MR (2006). Estimates of quality and reliability with the physiotherapy evidence-based database scale to assess the methodology of randomized controlled trials of pharmacological and nonpharmacological interventions. Phys Ther.

[28] de Morton NA (2009). The PEDro scale is a valid measure of the methodological quality of clinical trials: a demographic study. Aust J Physiother.

[29] Maher CG, Sherrington C, Herbert RD, Moseley AM, Elkins M (2003). Reliability of the PEDro scale for rating quality of randomized controlled trials. Phys Ther.

[30] Verhagen AP, de Vet HC, de Bie RA, Kessels AG, Boers M, Bouter LM, Knipschild PG (1998). The Delphi list: a criteria list for quality assessment of randomized clinical trials for conducting systematic reviews developed by Delphi consensus. J Clin Epidemiol.

[31] McCrary JM, Ackermann BJ, Halaki M (2015). A systematic review of the effects of upper body warm-up on performance and injury. Br J Sports Med.

[32] Aarts S, van den Akker M, Winkens B (2014). The importance of effect sizes. Eur J Gen Pract.

[33] Borenstein M (2023). How to understand and report heterogeneity in a meta-analysis: the difference between I-squared and prediction intervals. Integr Med Res.

[34] Herring LY, Dallosso H, Chatterjee S (2018). Physical Activity after Cardiac EventS (PACES) - a group education programme with subsequent text-message support designed to increase physical activity in individuals with diagnosed coronary heart disease: study protocol for a randomised controlled trial. Trials.

[35] Brouwers RW, van der Poort EK, Kemps HM, van den Akker-van Marle ME, Kraal JJ (2021). Cost-effectiveness of cardiac telerehabilitation with relapse prevention for the treatment of patients with coronary artery disease in the Netherlands. JAMA Netw Open.

[36] Batalik L, Dosbaba F, Hartman M, Konecny V, Batalikova K, Spinar J (2021). Long-term exercise effects after cardiac telerehabilitation in patients with coronary artery disease: 1-year follow-up results of the randomized study. Eur J Phys Rehabil Med.

[37] Bernal-Jiménez MÁ, Calle G, Gutiérrez Barrios A (2024). Effectiveness of an Interactive mHealth App (EVITE) in improving lifestyle after a coronary event: randomized controlled trial. JMIR Mhealth Uhealth.

[38] Redfern J, Singleton AC, Raeside R (2024). Integrated Text Messaging (ITM) for people attending cardiac and pulmonary rehabilitation: a multicentre randomised controlled trial. Ann Phys Rehabil Med.

[39] Avila A, Claes J, Buys R, Azzawi M, Vanhees L, Cornelissen V (2020). Home-based exercise with telemonitoring guidance in patients with coronary artery disease: does it improve long-term physical fitness?. Eur J Prev Cardiol.

[40] Su JJ, Yu DS (2021). Effects of a nurse-led eHealth cardiac rehabilitation programme on health outcomes of patients with coronary heart disease: a randomised controlled trial. Int J Nurs Stud.

[41] Widmer RJ, Allison TG, Lennon R, Lopez-Jimenez F, Lerman LO, Lerman A (2017). Digital health intervention during cardiac rehabilitation: a randomized controlled trial. Am Heart J.

[42] Maddison R, Rawstorn JC, Stewart RA (2019). Effects and costs of real-time cardiac telerehabilitation: randomised controlled non-inferiority trial. Heart.

[43] Snoek JA, Meindersma EP, Prins LF (2021). The sustained effects of extending cardiac rehabilitation with a six-month telemonitoring and telecoaching programme on fitness, quality of life, cardiovascular risk factors and care utilisation in CAD patients: the TeleCaRe study. J Telemed Telecare.

[44] Sankaran S, Dendale P, Coninx K (2019). Evaluating the impact of the HeartHab app on motivation, physical activity, quality of life, and risk factors of coronary artery disease patients: multidisciplinary crossover study. JMIR Mhealth Uhealth.

[45] Frederix I, Hansen D, Coninx K (2016). Effect of comprehensive cardiac telerehabilitation on one-year cardiovascular rehospitalization rate, medical costs and quality of life: a cost-effectiveness analysis. Eur J Prev Cardiol.

[46] Frederix I, Hansen D, Coninx K (2015). Medium-term effectiveness of a comprehensive internet-based and patient-specific telerehabilitation program with text messaging support for cardiac patients: randomized controlled trial. J Med Internet Res.

[47] Seron P, Oliveros MJ, Marzuca-Nassr GN (2024). Hybrid cardiac rehabilitation program in a low-resource setting: a randomized clinical trial. JAMA Netw Open.

[48] Mamataz T, Ghisi GL, Pakosh M, Grace SL (2021). Nature, availability, and utilization of women-focused cardiac rehabilitation: a systematic review. BMC Cardiovasc Disord.

[49] Oosenbrug E, Marinho RP, Zhang J, Marzolini S, Colella TJ, Pakosh M, Grace SL (2016). Sex differences in cardiac rehabilitation adherence: a meta-analysis. Can J Cardiol.

[50] Resurrección DM, Motrico E, Rigabert A, Rubio-Valera M, Conejo-Cerón S, Pastor L, Moreno-Peral P (2017). Barriers for nonparticipation and dropout of women in cardiac rehabilitation programs: a systematic review. J Womens Health (Larchmt).

[51] Andraos C, Arthur HM, Oh P, Chessex C, Brister S, Grace SL (2015). Women's preferences for cardiac rehabilitation program model: a randomized controlled trial. Eur J Prev Cardiol.

[52] Griffo R, Ambrosetti M, Furgi G (2012). Standards and outcome measures in cardiovascular rehabilitation. Position paper GICR/IACPR (Article in Italian). Monaldi Arch Chest Dis.

[53] Hwang R, Bruning J, Morris N, Mandrusiak A, Russell T (2015). A Systematic Review of the Effects of Telerehabilitation in Patients With Cardiopulmonary Diseases. J Cardiopulm Rehabil Prev.

[54] Dalal HM, Taylor RS (2016). Telehealth technologies could improve suboptimal rates of participation in cardiac rehabilitation. Heart.

[55] Wu C, Li Y, Chen J (2018). Hybrid versus traditional cardiac rehabilitation models: a systematic review and meta-analysis. Kardiol Pol.

[56] Ramachandran HJ, Jiang Y, Tam WW, Yeo TJ, Wang W (2022). Effectiveness of home-based cardiac telerehabilitation as an alternative to Phase 2 cardiac rehabilitation of coronary heart disease: a systematic review and meta-analysis. Eur J Prev Cardiol.

[57] Braver J, Marwick TH, Oldenburg B, Issaka A, Carrington MJ (2023). Digital health programs to reduce readmissions in coronary artery disease: a systematic review and meta-analysis. JACC Adv.

[58] Peretti A, Amenta F, Tayebati SK, Nittari G, Mahdi SS (2017). Telerehabilitation: review of the state-of-the-art and areas of application. JMIR Rehabil Assist Technol.

